# Nationwide surveillance in Thailand revealed genotype-dependent dissemination of carbapenem-resistant *Enterobacterales*

**DOI:** 10.1099/mgen.0.000797

**Published:** 2022-04-19

**Authors:** Dan Takeuchi, Anusak Kerdsin, Yukihiro Akeda, Yo Sugawara, Noriko Sakamoto, Yuki Matsumoto, Daisuke Motooka, Takuma Ishihara, Isao Nishi, Warawut Laolerd, Pitak Santanirand, Norihisa Yamamoto, Kazunori Tomono, Shigeyuki Hamada

**Affiliations:** ^1^​ Japan-Thailand Research Collaboration Center, Research Institute for Microbial Diseases, Osaka University, Suita, Japan; ^2^​ Faculty of Public Health, Kasetsart University Chalermphrakiat Sakon Nakhon Province Campus, Sakon Nakhon, Thailand; ^3^​ Department of Infection Metagenomics, Research Institute for Microbial Diseases, Osaka University, Suita, Japan; ^4^​ Innovative and Clinical Research Promotion Center, Gifu University Hospital, Gifu, Japan; ^5^​ Division of Infection Control and Prevention, Osaka University Hospital, Suita, Japan; ^6^​ Faculty of Medicine Ramathibodi Hospital, Mahidol University, Bangkok, Thailand

**Keywords:** carbapenem-resistant *Enterobacterales*, whole-genome sequencing, *bla*_NDM_ *bla*_OXA_, sequence type, Thailand

## Abstract

Carbapenem-resistant *Enterobacterales* (CRE) are a serious public health threat because of their rapid dissemination. To determine the epidemiological and genetic characteristics of CRE infections in Thailand, we performed whole-genome sequencing of 577 carbapenem-resistant *Klebsiella pneumoniae* isolates and 170 carbapenem-resistant *Escherichia coli* isolates from hospitals across the nation. The four most prevalent carbapenemase genes harboured by these bacteria were *bla*_NDM-1_, *bla*_NDM-5_, *bla*_OXA-181_ and *bla*_OXA-232_. The gene *bla*_NDM-1_ was identified in diverse sequence types. The gene *bla*_NDM-5_ was identified almost exclusively in *E. coli *. The genes *bla*_OXA-181_, *bla*_OXA-232_, and co-carriage of *bla*_NDM-1_ and *bla*_OXA-232_ were found in specific sequence types from certain provinces. Replicon typing revealed the diverse backbones of *bla*_NDM-1_- and *bla*_NDM-5_-harbouring plasmids and successful expansion of *bla*_NDM-1_-harbouring IncN2-type plasmids. Core-genome single-nucleotide polymorphism analysis suggested that *bla*_OXA-181_-, *bla*_OXA-232_-, *bla*_NDM-5_-, and co-carriage of *bla*_NDM-1_ and *bla*_OXA-232_-associated sub-clonal lineages have recently predominated in the provinces from where these isolates were isolated. Thus, we demonstrate genotype-dependent dissemination of CRE in Thailand, which is helpful for establishing infection-control strategies in CRE-endemic areas.

## Data Summary

**Impact Statement**

The spread of carbapenem-resistant *Enterobacterales* (CRE) poses a threat in clinical settings. Acquisition of carbapenemase is considered as a key mechanism of carbapenem-resistance. This article provides an overview of the genomic epidemiology of CRE nationwide in Thailand, where information has been limited. We highlight the prevalence of carbapenemase genotypes and replicon types of carbapenemase gene-harbouring plasmids, as well as their relationship with the genomic background of host bacteria. This article provides information helpful for establishing infection-control measures in CRE endemic areas such as Thailand.

Supplementary materials are deposited in the Microbiology Society’s data repository figshare account https://doi.org/10.6084/m9.figshare.16726060 [1].

## Introduction

Following the development of imipenem in 1985, carbapenems have long been used to treat infections caused by cephalosporin-resistant Gram-negative organisms [2]. Since the mid-2000s, carbapenem-resistant *Enterobacterales* (CRE) have spread globally, causing severe nosocomial infections for which treatment options are limited [3]. Hence, the spread of CRE has become a major public health threat necessitating urgent action.

Carbapenem-resistance is mediated by the acquisition of hydrolytic enzymes, loss of outer membrane porins, and/or overexpression of genes encoding efflux pumps [4]. Acquisition of hydrolytic carbapenemase enzymes is considered as a key mechanism of carbapenem-resistance. Carbapenemases hydrolyse carbapenems and other beta-lactams, and several of these enzymes such as New Delhi metallo-β-lactamase (NDM), oxacillin-hydrolysing enzyme (OXA), imipenemase (IMP), Verona integron-encoded metallo-β-lactamase (VIM), and *Klebsiella pneumoniae* carbapenemase (KPC) have been found worldwide [3]. Carbapenemase genes can be transferred by plasmids among various *Enterobacterales* species. Plasmid acquisition can then confer carbapenem-resistance in previously susceptible bacteria, making horizontal gene transfer the driving force behind the spread of CRE [5–7]. In addition, successful sequence-type (ST) lineages, such as clonal complex 258 (CC258) *K. pneumoniae* harbouring *bla*_KPC-2_, *bla*_KPC-3_ and *bla*_NDM-1_, have spread worldwide, leading to nosocomial outbreaks in several countries [8, 9] and indicating that bacterial clonal expansion of a specific ST is another important factor underlying its dissemination.

Southeast Asia is considered an endemic region for CRE [10–15]. The prevalence of CRE in Thailand was suggested in a recent report [16], which described the spread of carbapenemase-producing *K. pneumoniae* and *Escherichia coli* harbouring *bla*_NDM_ and *bla*_OXA-48_-like genes in five provinces. However, the genomic structure of CRE, including host bacterial ST, alleles of carbapenemase genes, plasmid replicon types and relationships among them remain unclear. Therefore, in this study, we investigated the epidemiological and genomic characteristics of CRE from 11 provinces in Thailand using whole-genome sequencing (WGS) to understand the evolutionary dynamics of CRE in this region from 2012 to 2017.

## Methods

### Isolation of CRE

The surveillance network consisted of 11 hospitals in 11 provinces, representing the northern (Phayao), western (Tak), north-eastern (Bueng Kan, Udon Thani, Sakon Nakhon, Nakhon Phanom, and Mukdahan), central (Surin and Bangkok) and southern (Chumphon and Surat Thani) regions of Thailand. The surveillance periods differed depending on the provinces (Table S1, available in the online version of this article). Isolates were obtained from various clinical specimens of 744 patients (e.g. blood, sputum, urine, abdominal fluid and stool). All isolates were subjected to bacterial identification and antimicrobial susceptibility testing at hospitals where they were isolated, and all carbapenem-resistant *Enterobacterales* were collected. In Bangkok, carbapenem-resistant *K. pneumoniae* and *E. coli* isolates were collected. All collected isolates were further analysed. Bacterial identification and antimicrobial susceptibility testing were re-examined as follows. Bacterial species were identified using matrix-assisted laser desorption/ionisation time-of-flight mass spectrometry (MALDI Sepsityper; Bruker Daltonics, Bremen, Germany) and the API 20E system (bioMérieux, Marcy l’Étoile, France). Antimicrobial resistance was examined using the broth microdilution method with the MicroScan Walkaway Plus system (Beckman Coulter, Brea, CA, USA) and EIKEN dry plate (Eiken, Tokyo, Japan). MIC breakpoints were defined according to the standards established by the Clinical and Laboratory Standards Institute (M100-S22) [17]. The MIC of colistin referred to the criteria of non-fermenting Gram-negative rod bacteria other than *Pseudomonas aeruginosa *, *Acinetobacter* spp., *Burkholderia cepacia* and *Stenotrophomonas maltophilia *. Carbapenem-resistant *K. pneumoniae* and *E. coli* isolates were subjected to WGS, and the isolates of other species were subjected to PCR to detect carbapenemase genes. The sequences of the PCR primers used in this study are summarized in Table S2. Carbapenem-resistant isolates with carbapenemase genes were defined as CRE. When the same ST strains were isolated from a patient at different time points, the strain isolated first was selected for further analysis to avoid duplication of the clone.

### Whole-genome sequencing

Bacterial isolates were cultured in Brain Heart Infusion broth (BD Biosciences, Franklin Lakes, NJ, USA) overnight and whole-cell DNA was extracted using a DNeasy PowerSoil Kit (Qiagen, Hilden, Germany). WGS was performed using a HiSeq 3000 (Illumina, San Diego, CA, USA), PacBio RSII (Pacific Biosciences, Menlo Park, CA, USA), and/or GridION (Oxford Nanopore Technologies, Oxford, UK) with R9.4 flow cells. The genomic DNA library for HiSeq and PacBio was prepared as described previously [15], whereas that for GridION was prepared using the Ligation Sequencing Kit 1D and Native Barcoding Kit 1D (Oxford Nanopore Technologies).

Allowing duplication, 697 and 68 isolates were subjected to HiSeq and PacBio RSII/GridION sequencing, respectively. Among the 68 isolates subjected to long-read sequencing, 57 and 11 isolates were sequenced using the PacBio RSII and GridION, respectively. Isolates for PacBio RSII were selected on a first-come basis. Isolates for GridION were chosen from those sequenced using the HiSeq in advance, which were diverse in terms of species, sequence types, carbapenemase genotypes and provinces from where they were isolated. Information on the WGS sequence data for each isolate is summarized in Table S3.

### *De novo* assembly of WGS sequence data

WGS data were subjected to *de novo* assembly. HiSeq reads were assembled using CLC Genomics Workbench 11.0.1 (CLC Bio, Aarhus, Denmark) after trimming adapter sequences and removing low-quality reads with a threshold of quality score <30. PacBio RSII reads were assembled using HGAP 3 with pre-assembly (settings; subread length >500 bp, polymerase read quality >0.8, polymerase read length >100 bp) and Quiver polishing. GridION reads were co-assembled with HiSeq reads using unicycler version 0.4.4 [18] with default settings.

### Confirmation of STs, carbapenemase genotypes and plasmid replicon types

Assembled contigs were subjected to the batch uploader of the CGE Bacterial Analysis Pipeline to confirm the STs using MLST 1.6 [19] and detect carbapenemase genes with ResFinder 2.1 [20] using default settings. Contigs of isolates with undetermined ST and/or carbapenemase genotype were further subjected to MLST 2.1 and ResFinder 4.1, individually. The *E. coli* MLST scheme used the following seven housekeeping genes: *adk*, *fumC*, *gyrB*, *icd*, *mdh*, *purA* and *recA*. The new STs of *K. pneumoniae* and *E. coli* were submitted to the BIGSdb-Pasteur databases (http://bigsdb.pasteur.fr/) and PubMLST (https://pubmlst.org/), respectively. The replicon types of plasmids were confirmed using PlasmidFinder 2.1 [21].

### Calculation of diversity index

Simpson’s diversity index [22] was used to evaluate the geographical and population diversity of the carbapenemase genotype [23] and was calculated as follows:

,$$D=1-⟮\frac{\sum_{i=1}^{R}n_{i}(n_{i}-1)}{N(N-1)}⟯$$

where $n_{i}$ is the number of isolates with each sequence type i among the carbapenemase genotypes, N is the total number of isolates with carbapenemase genotypes, and R is the total number of STs in the carbapenemase genotypes. The value of Simpson’s diversity index, D, ranges from 0 to 1, with 0 representing no diversity and 1 representing infinite diversity. The confidence interval was calculated using the approximation formula. The bootstrap mean and confidence interval of Simpson’s diversity index were also calculated for the dataset of *bla*_NDM-5_-carrying *K. pneumoniae* isolates using 10 000 bootstrap samples. All calculations were performed using R version 3.6.2 (www.r-project.org).

### Reconstruction of plasmid sequence *in silico* and replicon typing

Plasmid sequences from long-read sequencers were reconstructed by the circularization of assembled contigs. Contigs from PacBio were circularized at the beginning of the duplicated sequence if they met the criterion of similarity of >99 % over 5000 bp. Contigs from the hybrid assembly of GridION and HiSeq were circularized using unicycler version 0.4.4 [18] through the assembly steps. The presence of the carbapenemase gene was confirmed for all circularized plasmid sequences.

Plasmid sequences from the HiSeq sequencing data were determined from assembled contigs using MOB-recon module in MOB-suite [24]. The software tool extracted plasmid-associated contigs, providing clusters of contigs of plasmid origin. After clustering, clusters containing a carbapenemase-gene sequence were extracted and used as carbapenemase-harbouring plasmid sequences for further characterisation.

To infer the replicon types of plasmids harbouring carbapenemase genes of interest, successfully circularized contigs from long-read sequencing data, carbapenemase gene-containing non-circularized contigs from long-read sequencing data, and estimated plasmids sequences by MOB-suite from short-read sequencing data were submitted to PlasmidFinder 2.1.

### Core-genome SNP analysis and evaluation of sub-clonal clade

Core-genome phylogenetic analysis was performed for representative STs as described previously [25]. Briefly, assembled contigs were aligned using progressiveMauve [26], and recombination sites were removed using Gubbins [27] (parameters: ‘--tree_builder raxml’). After single-nucleotide polymorphisms (SNPs) were extracted using SNP-sites [28], a phylogenetic tree was constructed using FastTree [29] in a generalized time-reversible model and visualized using iTol [30]. Based on the phylogenetic trees, sub-clonal clades were evaluated based on the pair-wise SNP distance of the constituent isolates by using the output files from SNP-sites. According to previous reports, an SNP distance ≤21 suggested clonality of *K. pneumoniae* [31, 32], whereas an SNP distance ≤40 was considered as a clonal group in *E. coli* ST 410 [33].

## Results

### Geographical distribution and CRE species

**Fig. 1.**
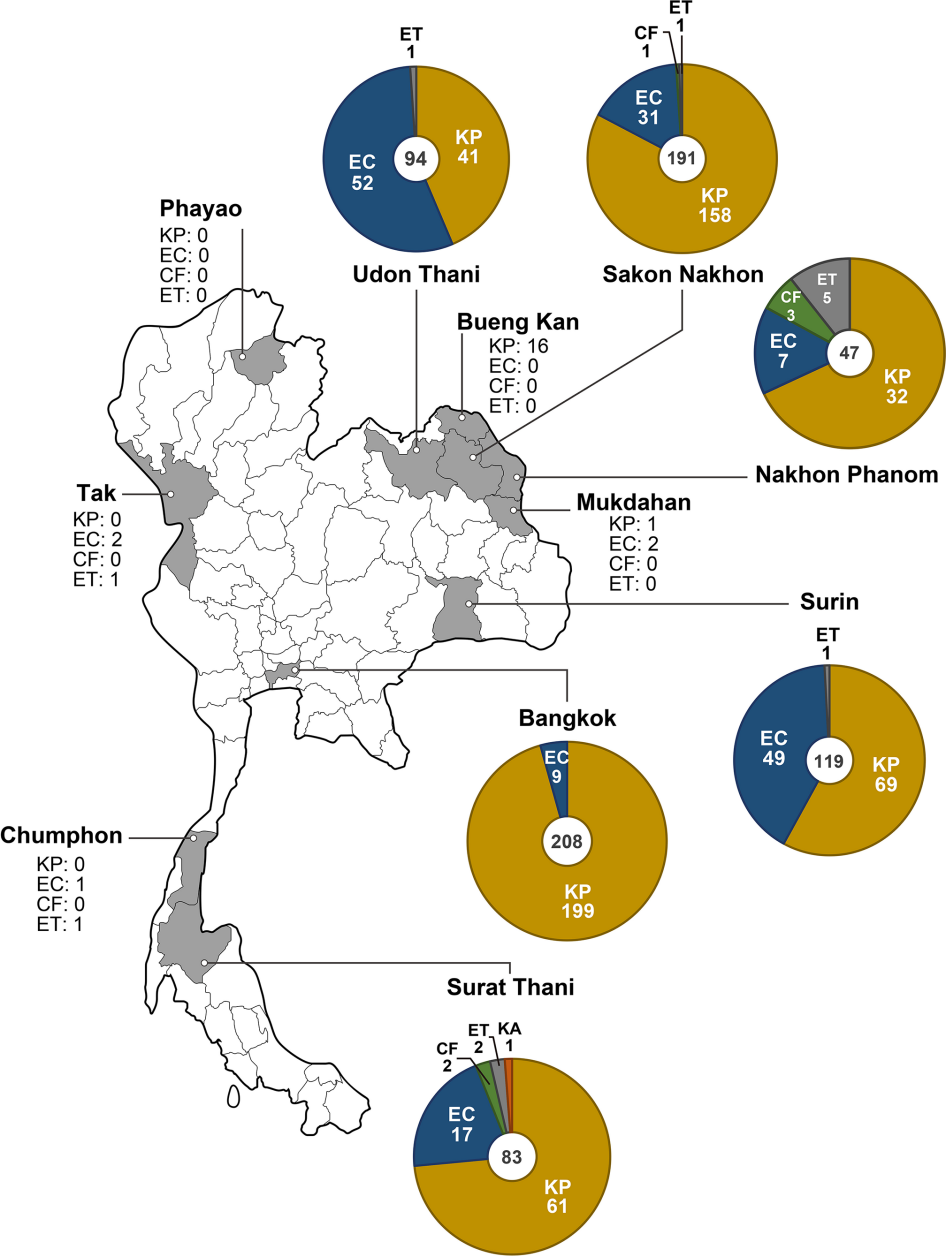
Geographical distribution of carbapenem-resistant *Enterobacterales* with carbapenemase genes. The isolation ratio of all species is shown on the map of Thailand. The total number of isolates is shown in the centre of the pie chart, and the number of each species is mentioned next to the species name. Please note that the ratio in Bangkok does not represent the isolation ratio of all CRE because of the difference in sampling methods as described in the Methods section. KP: *Klebsiella pneumoniae *, EC: *Escherichia coli *, CF: *Citrobacter freundii *, ET: *Enterobacter cloacae *, and KA: *Klebsiella aerogenes *.

In total, 766 isolates from CRE-infected patients were collected from 11 selected provinces. Among them, 96.9 % were from six provinces – Udon Thani, Sakon Nakhon, Nakhon Phanom, Surin, Bangkok and Surat Thani – which are in north-eastern, central and southern Thailand (Fig. 1). *K. pneumoniae* (577 isolates) and *E. coli* (170 isolates) accounted for 97.5 % of all CRE isolates. Although the isolation of *Enterobacter cloacae *, *Citrobacter freundii* and *Klebsiella aerogenes* was rare, these species were found in multiple provinces, including those with low isolation rates of CRE. *K. pneumoniae* was the most frequently isolated species in all provinces, except for in Udon Thani, where the isolation ratio of *E. coli* was higher. *K. pneumoniae* and *E. coli* were frequently isolated from urine (284 specimens including urine from catheter) and sputum (283 specimens), followed by blood (71 specimens). Ten isolates were isolated from screening of rectal swabs. All isolates of two predominant species, *K. pneumoniae* and *E. coli *, were further analysed using WGS.

### STs and carbapenemase genotype

**Fig. 2.**
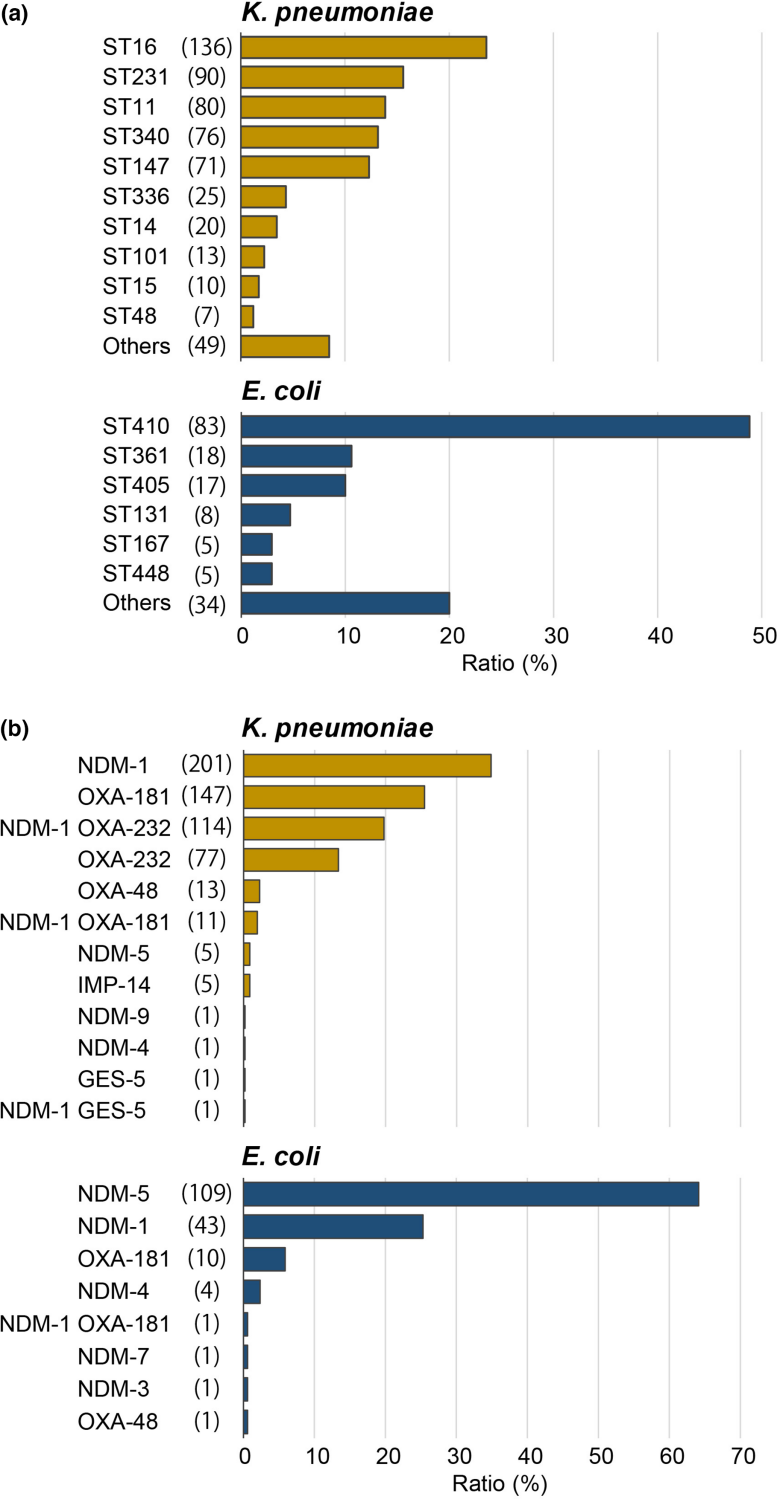
Distribution of STs (a) and carbapenemase genes (b) in *K. pneumoniae* and *E. coli* isolates. The number of isolates is mentioned in parentheses.

The STs of both species were remarkably diverse (Fig. 2a). In total, 43 and 26 STs were identified in *K. pneumoniae* and *E. coli *, respectively, including newly identified ST5808 and ST5819 of *K. pneumoniae* and ST10210 of *E. coli *. The most frequent type identified in *K. pneumoniae* was ST16, followed by ST231, ST11, ST340 and ST147. For *E. coli *, ST410 comprised the majority of isolates, followed by ST361, ST405 and ST131.

Various carbapenemase genes were identified, including *bla*_NDM-1_, *bla*_NDM-5_, *bla*_OXA-181_ and *bla*_OXA-232_ (Fig. 2b). The gene *bla*_NDM-5_ was predominant in *E. coli *, whereas *bla*_OXA-181_ and *bla*_OXA-232_ were predominant in *K. pneumoniae *. Co-carriage of *bla*_NDM-1_ and *bla*_OXA-232_ was the third most frequent genotype in *K. pneumoniae *. The isolates of five major carbapenemase genotypes, *bla*_NDM-1_, *bla*_NDM-5_, *bla*_OXA-181_, *bla*_OXA-232_ and co-carriage of *bla*_NDM-1_ and *bla*_OXA-232_ were further characterized.

### Antimicrobial resistance

The antibiogram of both species showed high proportions of resistance to a wide range of beta-lactams (Fig. S1). Notably, the carbapenem antibiotic biapenem exerted antibiotic effects against 68 % of *E. coli* isolates. Aminoglycosides were generally effective against 60–80 % of isolates of both species. Colistin exhibited antimicrobial activity against 73 and 100 % of *K. pneumoniae* and *E. coli* isolates, respectively.

The antibiogram was slightly different in the context of the carbapenemase genotype (Fig. S2). Around 40 and 60 % of isolates with *bla*_OXA-181_ were susceptible to cefmetazole and flomoxef, respectively, and the rates were higher than those of isolates with other genes. Isolates with *bla*_OXA-181_ also showed slightly higher rates of susceptibility to carbapenems compared to other isolates. Biapenem was mostly efficacious towards isolates with *bla*_NDM-5_, reflecting an association between this gene and antibiotic susceptibility in *E. coli *. Isolates with *bla*_OXA-232_ and co-carriage of *bla*_NDM-1_ and *bla*_OXA-232_ showed limited susceptibility to aminoglycosides.

### Geographical distribution of carbapenemase genes

**Fig. 3.**
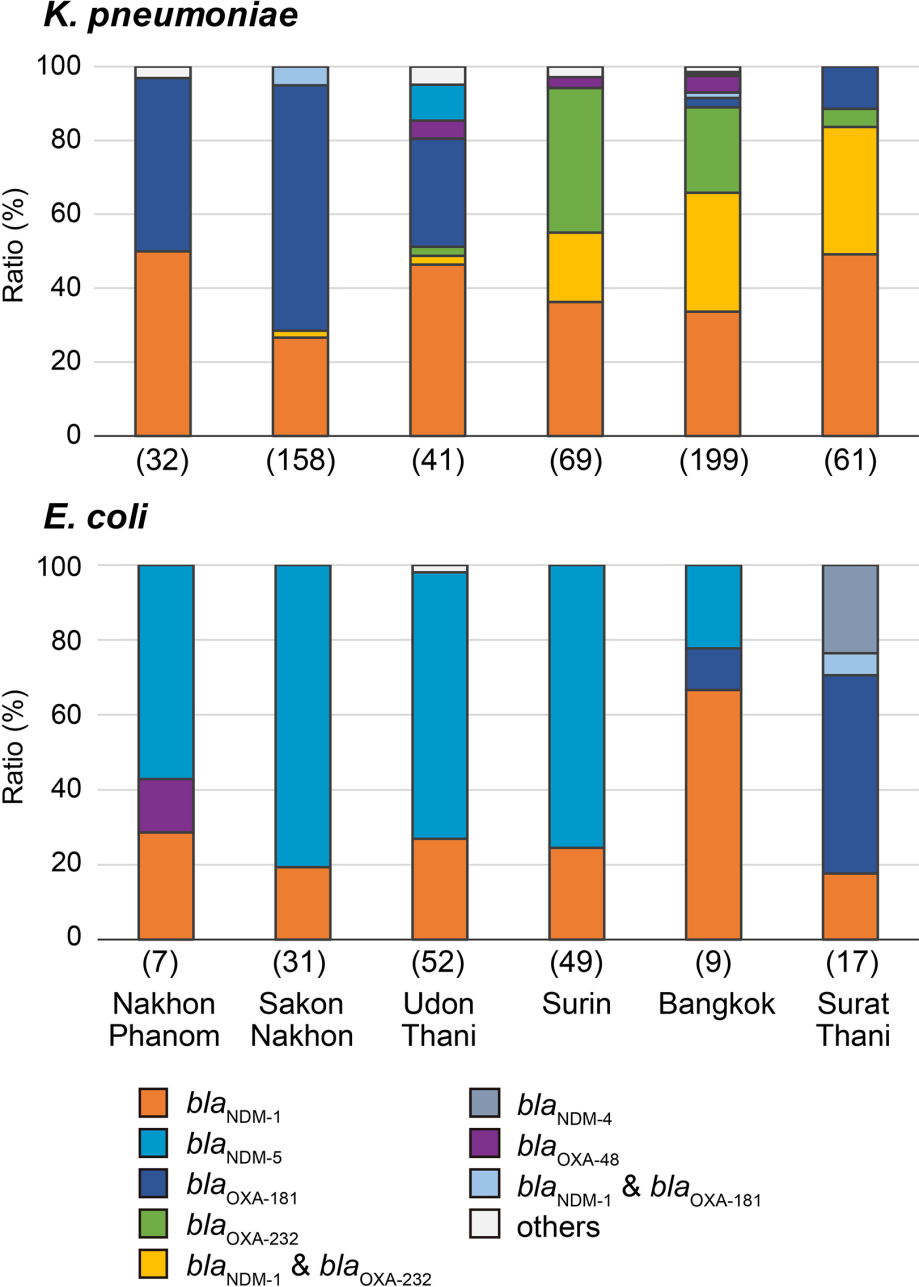
Distribution of carbapenemase genes among six representative provinces. The number of isolates in each province is shown in parentheses.

The distribution of carbapenemase genes in representative provinces is summarized in Figs 3 and S3. The gene *bla*_NDM-1_ was widely distributed among all provinces, except for in Phayao, Tak and Chumphon, where the number of isolates was too small to evaluate the extent of endemicity (Fig. S3). Although *bla*_NDM-5_ was also widespread, the isolation ratio decreased towards the south (Fig. 3). The genes *bla*_OXA-181_ and *bla*_OXA-232_ appeared to be distributed in different areas, particularly for *K. pneumoniae *. Isolates harbouring *bla*_OXA-181_ were frequently isolated from the north-eastern contiguous provinces of Nakhon Phanom, Sakon Nakhon and Udon Thani, whereas *bla*_OXA-232_-harbouring isolates were mainly found in the central provinces of Surin and Bangkok. The distribution of *bla*_NDM-1_ and *bla*_OXA-232_ co-harbouring isolates resembled that of *bla*_OXA-232_, except that co-harbouring isolates were more frequently found in the southern province of Surat Thani and north-eastern province of Bueng Kan. The prevalence of co-harbouring isolates was high in Bueng Kan, despite their low prevalence in the neighbouring provinces of Nakhon Phanom, Sakon Nakhon and Udon Thani.

The prevalence of carbapenemase genes was compared based on Simpson’s diversity index (Table S4; geographical diversity). The gene *bla*_NDM-1_ showed the greatest diversity in terms of its distribution among provinces, with a diversity index of 0.81 and without overlap in confidence intervals with the other genes, followed by *bla*_NDM-5_ (0.72). The diversity of the *bla*_OXA-181_ and *bla*_OXA-232_ distribution was relatively low, with a diversity index of 0.53.

### Distribution of carbapenemase genes among STs

The distribution of carbapenemase genes among host bacterial STs also differed depending on the carbapenemase genotype (Table S4; diversity of STs). The gene *bla*_NDM-1_ was identified in 35 of 43 STs in *K. pneumoniae* and 19 of 26 STs in *E. coli* and was often carried by rare *E. coli* STs (Table S5). The gene *bla*_NDM-5_ was preferentially found in *E. coli* STs, such as ST410, ST361 and ST405. In contrast, the distribution of *bla*_OXA-181_ and *bla*_OXA-232_ was relatively limited in *K. pneumoniae* STs. The gene *bla*_OXA-181_ was mostly found in ST11 and ST340, and *bla*_OXA-232_ was prevalent in ST231. Co-carriage of *bla*_NDM-1_ and *bla*_OXA-232_ was mostly limited to ST16 of *K. pneumoniae *. The diversity index indicated that *bla*_NDM-1_ was the most diverse in terms of host bacterial STs in both *K. pneumoniae* (diversity index=0.88) and *E. coli* STs (0.92), whereas *bla*_OXA-181_, *bla*_OXA-232_ and co-carriage of *bla*_NDM-1_ and *bla*_OXA-232_ were less diverse, with lower diversity indices.

### Replicon types of carbapenemase-harbouring plasmids

The replicon types of plasmids harbouring carbapenemase genes of interest were classified. In total, 777 plasmids sequences were obtained from long-read and short-read sequencing data (623 and 154 sequences derived from *K. pneumoniae* and *E. coli *, respectively), consisting of successfully circularized contigs from long-read sequencing data (51 sequences), carbapenemase gene-containing non-circularized contigs from long-read sequencing data (12 sequences), and estimated plasmids sequences by MOB-suite from short-read sequencing data (714 sets of sequences). Among them, the replicon types of 609 plasmid sequences could be determined (469 and 140 sequences derived from *K. pneumoniae* and *E. coli *, respectively), and the remaining 168 sequences failed to be inferred.

**Fig. 4.**
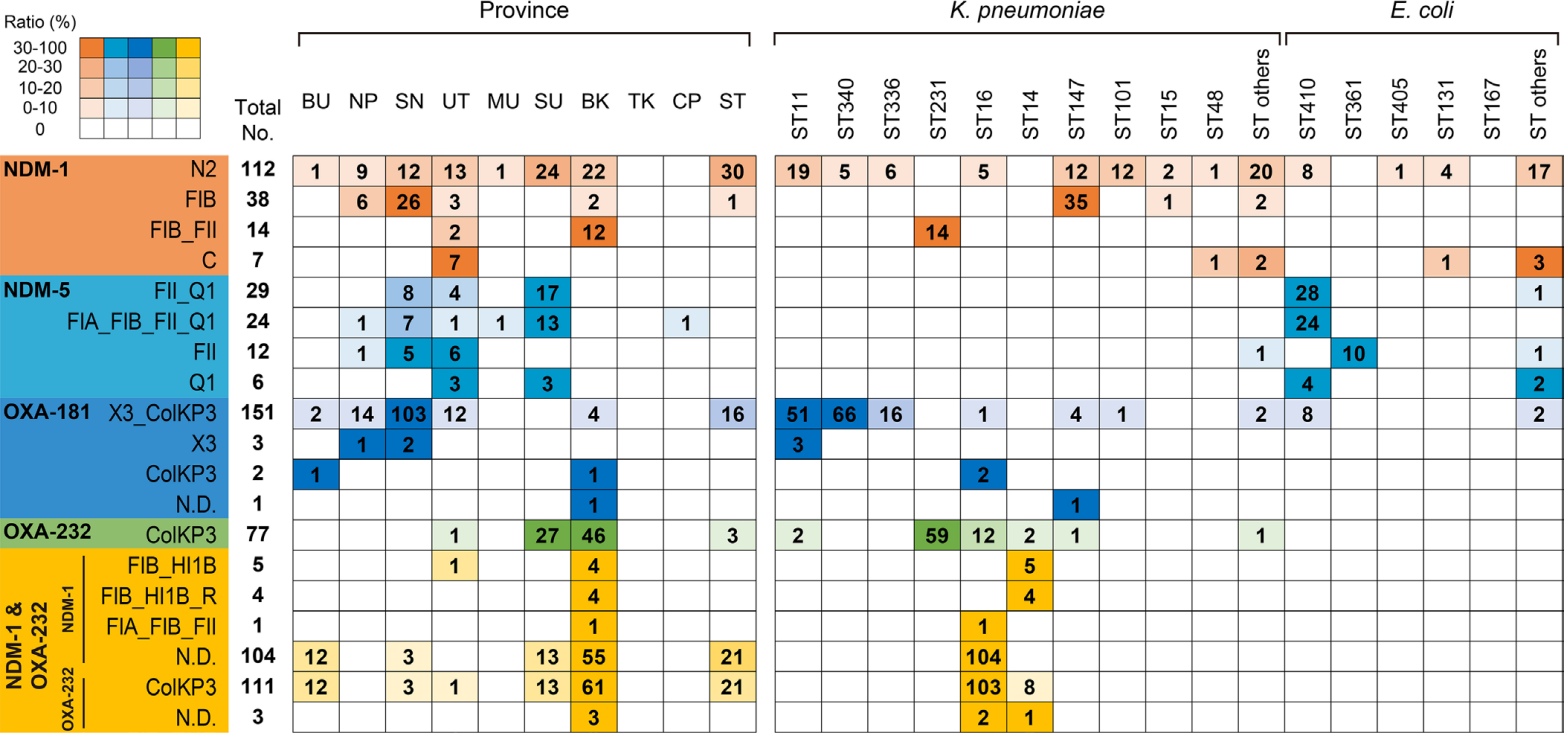
Geographical and sequence type-based distribution of representative plasmid replicon types in isolates with each genotype. Each coloured cell represents the ratio of the total number of plasmids of the same replicon type. N.D., not determined. BU: Bueng Kan, NP: Nakhon Phanom, SN: Sakon Nakhon, UT: Udon Thani, MU: Mukdahan, SU: Surin, BK: Bangkok, TK: Tak, CP: Chumphon and ST: Surat Thani.

The replicon types of *bla*_NDM-1_-harbouring plasmids were classified into various types, including single- and multi-replicons (Figs 4, S4 and S5). Among them, *bla*_NDM-1_-harbouring IncN2-type plasmids were broadly distributed across provinces (8 of 11) and STs (25 of 43 STs in *K. pneumoniae* and 17 of 26 STs in *E. coli *). *bla*_NDM-5_-harbouring plasmids also showed a variety of replicon types. In contrast to *bla*_NDM-1_ and *bla*_NDM-5_, the replicon types of *bla*_OXA-181_- and *bla*_OXA-232_-harbouring plasmids among the isolates with each gene were mostly consistent. Ninety-six percent (151 of 157 plasmids) and 100 % (77 of 77 plasmids) of *bla*_OXA-181_- and *bla*_OXA-232_-harbouring plasmids were the IncX3_ColKP3 and IncColKP3 types, respectively.

The replicon types of plasmids in co-carriers of *bla*_NDM-1_ and *bla*_OXA-232_ were inconsistent. Most *bla*_OXA-232_-harbouring plasmids in the co-carriers (111 of 114 plasmids) were successfully confirmed as the IncColKP3 type. In contrast, most replicon types of the *bla*_NDM-1_-harbouring plasmids in the co-carriers (104 of 114 plasmids) could not be determined by PlasmidFinder.

Representative circularized plasmid sequences obtained from long-read sequencers are summarized in Table S6. They were selected based on the replicon types, which were prevalent among isolates with each genotype (Figs 4, S4 and S5). Unfortunately, some *bla*_NDM-1_- and *bla*_NDM-5_-harbouring plasmids of the prevalent replicon types could not be obtained, as they were not sequenced with the long-read sequencer. IncX3_ColKP3 and IncX3 plasmids were selected for *bla*_OXA-181_- and *bla*_OXA-232_-harbouring plasmids, respectively, because they were predominant replicon types. The pKP164_NDM1 plasmid was selected as a representative sequence of *bla*_NDM-1_-harbouring plasmids in *K. pneumoniae* ST16 isolates co-carrying *bla*_NDM-1_ and *bla*_OXA-232_, which was considered as a successful clone.

### Core-genome SNP analysis of representative STs

**Fig. 5.**
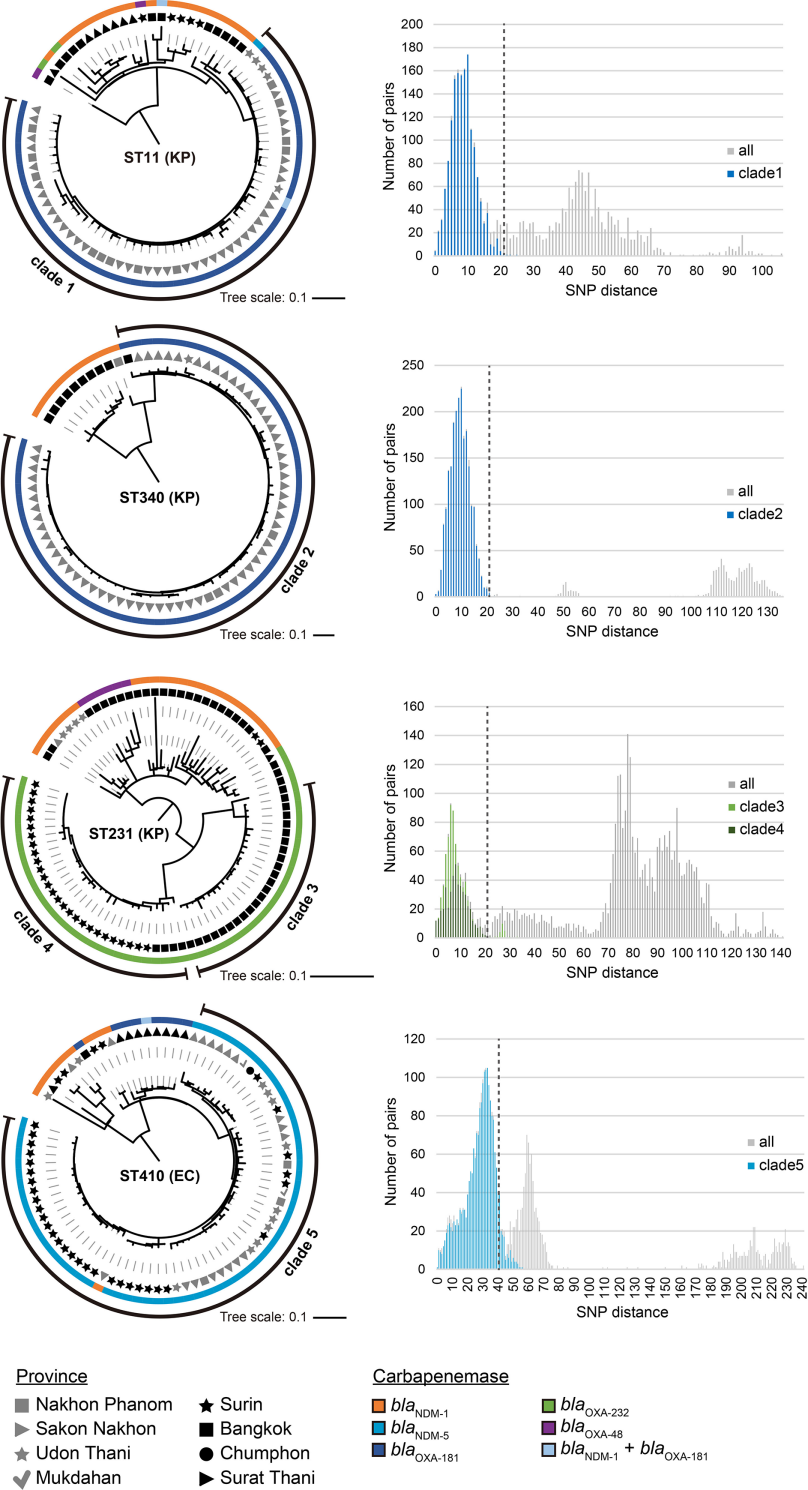
Phylogenetic analysis of ST11, ST340 and ST231 of *K. pneumoniae* (KP) and ST410 of *E. coli* (EC) isolates and the distribution of pair-wise SNP distance. Phylogenetic tree was based on chromosomal core-genome SNPs of constituent isolates of each ST (ST11 : 413 SNPs, ST340 : 319 SNPs, ST231 : 642 SNPs and ST410 : 809 SNPs). Scale bar indicates the number of nucleotide substitutions per site. Phylogenetic tree was drawn as a mid-rooted tree in a circular format, representing the carbapenemase genotype in relation to the province from where the isolates were obtained. Sub-clonal clades are indicated by a black strip in the outermost circle. The bar graph on the right side of each phylogenetic tree shows the distribution of pair-wise SNP distances in sub-clonal clades. Vertical dashed line represents the threshold of clonality for each species.

To evaluate the relationship between host bacterial evolution and the carbapenemase genotype, phylogenetic analysis of core-genome SNPs in representative STs (ST11, ST340, ST231 and ST16 of *K. pneumoniae* and ST410 of *E. coli *) was performed (Figs 5 and S6). Large clades were associated with *bla*_OXA-181_ in ST11/340 (clades 1 and 2 in Fig. 5), *bla*_OXA-232_ in ST231 (clades 3 and 4 in Fig. 5), *bla*_NDM-5_ in ST410 (clade 5 in Fig. 5), and co-carriers of *bla*_NDM-1_ and *bla*_OXA-232_ in ST16 (clades 6 and 7 in Fig. S6). All isolates in clade 3 were collected from Bangkok, whereas isolates in other clades were collected from different provinces.

The distribution of the pair-wise SNP distance in the clades was further analysed (Figs 5 and S6). SNP distances of isolates in clades 1–6 were smaller than those of all isolates, showing that most distances were within the clonality thresholds (clade 1 : 99.8%, clade 2 : 99.9%, clade 3 : 91.7%, clade 4 : 99.8%, clade 5 : 92.9% and clade 6 : 90.9%). The distances of isolates in clade 7 were widely distributed, as only 40.7 % were within the threshold (Fig. S6).

The replicon types of plasmids carried by clones in clades 1–6 were further analysed. Those of *bla*_OXA-181_- and *bla*_OXA-232_-harbouring plasmids in clades 1–4 were almost consistent. The replicon types of 93 % (51 of 55 plasmids) of *bla*_OXA-181_-harbouring plasmids in clade 1 and those of all *bla*_OXA-181_-harbouring plasmids in clade 2 were IncX3_ColKP3. All *bla*_OXA-232_-harbouring plasmids in clades 3 and 4 were ColKP3. The replicon types of *bla*_NDM-5_-harbouring plasmids in clade 5 were largely divided into two types, IncFII(pAMA1167-NDM-5)_IncQ1 (28 plasmids) and IncFIA_IncFIB(AP001918)_IncFII(pAMA1167-NDM-5)_IncQ1 (24 plasmids), which were dispersed in the clade (Fig. S7). The replicon types of all *bla*_NDM-1_-harbouring plasmids in co-carriers of *bla*_NDM-1_ and *bla*_OXA-232_ in clade 6 could not be determined, although those of all *bla*_OXA-232_-harbouring plasmids in co-carriers in this clade were ColKP3.

## Discussion

We determined the genetic epidemiology of CRE in Thailand. The spread of CRE was confirmed across Thailand, and carbapenemase genes were distributed via plasmid-mediated and bacterial clonal expansion. The most prevalent CRE species was *K. pneumoniae* followed by *E. coli *, which is in accordance with a previous report [16]. Other species, such as *E. cloacae *, *C. freundii* and *K. aerogenes *, were detected in several provinces, although the isolation of these species was relatively rare. Interestingly, the order of prevalence of CRE species appears to have the opposite trend in a neighbouring country, Myanmar, where *E. coli* is more prevalent than *K. pneumoniae* [13, 15]. Considering the association between *bla*_NDM-5_ and *E. coli* in our study and that *bla*_NDM-5_ is the most prevalent genotype in Myanmar, the prevalent genotype may influence the selection of CRE species.

*K. pneumoniae* and *E. coli* belonged to various STs. Most of these STs have been reported as successful lineages of CRE worldwide, including *K. pneumoniae* ST11, ST14, ST101 and ST147 [34] and *E. coli* ST131 and ST410 [33, 35]. Other STs, including *K. pneumoniae* ST231, ST340 and ST48 and *E. coli* ST405 and ST167, have been reported in various countries [13, 15, 36, 37]. *K. pneumoniae* ST340 and ST11 belong to CC258, indicating its association with global dissemination of CRE [34]. Given the presence of diverse CRE species and STs, Thailand is a possible reservoir area of CRE.

Among the carbapenemase genes identified, *bla*_NDM-1_ showed the broadest distribution in terms of geography and STs. Plasmid replicon typing revealed successful expansion of IncN2-type plasmids, accounting for the major distribution of *bla*_NDM-1_. IncN2-type plasmids constitute a subgroup of IncN plasmids [38] known for their conjugative properties and broad host range [39]. The *bla*_NDM-1_-harbouring IncN2-type plasmid was first identified in an *E. coli* strain isolated in Australia from a patient transferred from Bangladesh (p271A; GenBank accession no. JF785549.1) [38]. Since then, the plasmid has been reported in Thailand (pNDM_ECS01; GenBank accession no. KJ413946.1) [40] and China (pJN24NDM1; GenBank accession no. MK368725.1) [41]. A representative *bla*_NDM-1_-harbouring IncN2-type plasmid in this study (pC057_NDM1, Table S6) shared high sequence similarity with p271A (coverage: 87%, identity: 99.93%), pNDM_ECS01 (coverage: 100%, identity: 99.97%) and pJN24NDM1 (coverage: 100%, identity: 99.97%). The worldwide spread of similar plasmids suggests the role of IncN2-type plasmid as a carbapenemase gene transporter. In addition, various replicon types were found for *bla*_NDM-1_- and *bla*_NDM-5_-harbouring plasmids. The multiple replicons allow for a broader host range, thereby leading to spread of the gene.

Bacterial clonal expansion is a driving force for CRE dissemination. We found that the spread of *bla*_NDM-5_, *bla*_OXA-181_, *bla*_OXA-232_ and co-carriage of *bla*_NDM-1_ and *bla*_OXA-232_ was preferentially associated with clonal expansion of *E. coli* ST410, and *K. pneumoniae* ST11/340, ST231 and ST16, respectively. Phylogenetic analysis revealed an association of genotypes with sub-clonal lineages (clades 1–6 in Figs 5 and S6). The lineages associated with *bla*_OXA-181_, *bla*_OXA-232_, *bla*_NDM-5_, and co-carriage of *bla*_NDM-1_ and *bla*_OXA-232_ showed high clonality and inter-regional dissemination, revealing the recent evolution and expansion across provinces. The movement of people across these provinces may have been a factor promoting their dissemination. CRE screening of newly hospitalized patients with risk factors, such as a recent history of hospitalization, may be an effective infection-control measure. Furthermore, almost consistent replicon types in *bla*_OXA-181_- and *bla*_OXA-232_-harbouring plasmids in *bla*_OXA-181_ and *bla*_OXA-232_-associated *K. pneumoniae* ST11/340 and ST231 lineages suggested expansion of the lineages after plasmid acquisition of the plasmids. However, the presence of two different replicon types of plasmids in the *bla*_NDM-5_-associated *E. coli* ST410 lineage suggested that the isolates in this lineage acquired *bla*_NDM-5_-harbouring plasmids after expansion. In any case, the expansion of successful lineages promotes dissemination of CRE of specific STs.

Co-carriage of *bla*_NDM-1_ and *bla*_OXA-232_ was exclusively observed in *K. pneumoniae* ST16 and ST14. Previous studies suggested that the co-carriers frequently belonged to the same STs [42–45]. Furthermore, the replicon types of *bla*_OXA-232_-harbouring plasmids identified in this study were the same as those determined in previous studies [43–45]. A suitable combination of genetic background and plasmid replicon type may promote successful clonal dissemination of the co-carriers, although the molecular reasons for this require further analysis.

There were several limitations to this study. Although 11 provinces were enrolled, more distant provinces, particularly from the central part of Thailand, should have been included. The surveillance network was constructed upon the start of the study; obtaining sufficient information on CRE prevalence is required to generate a sustainable and representative network. The number of isolates obtained from Phayao, Mukdahan, Tak and Chumphon provinces was lower than that obtained from other provinces. Relatively shorter collection periods in these areas may have led to lower CRE prevalence rates (Table S1). Additionally, skewed sampling may have influenced the CRE species composition. The location of hospitals may also have influenced the number of isolates. Hospitals in the Phayao and Tak provinces are in mountain areas, away from the downtown area. The movement of people from other regions was restricted and, therefore, the influx of CRE from other regions may have been limited. In addition, hospitals in the Phayao, Tak and Chumphon provinces are small with 200–300 beds. The lower number of enrolled patients from these areas may have influenced the number of isolates. Further prospective studies are required to evaluate the biassed geographical spread of CRE. Although plasmids sequences were determined from assembled contigs from HiSeq sequencing data using MOB-suite, the software does not perform well for novel plasmids that do not share significant sequence similarity to those in the database [24]. This limitation likely led to the difficulty in estimating *bla*_NDM-1_-harbouring plasmids in *K. pneumoniae* isolates with co-carriage of *bla*_NDM-1_ and *bla*_OXA-232_. Furthermore, reconstructing plasmid sequences from short-read sequencing data remains challenging. In addition to the reconstruction method using MOB-suite, we also performed replicon typing of assembled contigs on which carbapenemase genes were carried. Among the 714 contigs, replicon types of 439 contigs (61.5%) could be determined. The number of contigs with successfully determined replicon types differed depending on the types of carbapenemase genes carried on the contigs. Although the replicon types of 95 % of *bla*_OXA-181_- and 98 % of *bla*_OXA-232_-carrying plasmids were successfully determined, those of only 37 % of *bla*_NDM-1_- and none of *bla*_NDM-5_-carrying plasmids were determined. In contrast, reconstruction using MOB-suite determined the replicon types of 68.8%, 85.9%, 100% and 98.4 % of *bla*_NDM-1_-, *bla*_NDM-5_-, *bla*_OXA-181_- and *bla*_OXA-232_-containing plasmid sequences, respectively. These points should be considered when interpreting the results for the replicon types.

We verified the nationwide prevalence of CRE in Thailand. The pattern of spread differed according to the carbapenemase genes. Plasmid expansion and bacterial clonal expansion were the two most important factors determining CRE dissemination. Particularly, the spread of IncN2-type plasmids contributed to dissemination of *bla*_NDM-1_. We also identified the successful endemic nature of ST11, ST340 and ST231 of *K. pneumoniae* and ST410 of *E. coli *. Continuous close monitoring of these endemic plasmids and clones is required.

## Supplementary Data

Supplementary material 1

Supplementary material 2
