# Supplementary material for: Nationwide surveillance in Thailand revealed genotype-dependent dissemination of carbapenem-resistant Enterobacterales
Source: Microb Genom. 2022 Apr 19;8(4):000797. doi: 10.1099/mgen.0.000797 (PMC9453063; doi:10.1099/mgen.0.000797)
Supplement: Supplementary material 1 [file mgen-8-0797-s001.pdf]

Supplementary Figure 1.

Antibiogram of carbapenem-resistant *K. pneumoniae* and *E. coli* isolates.

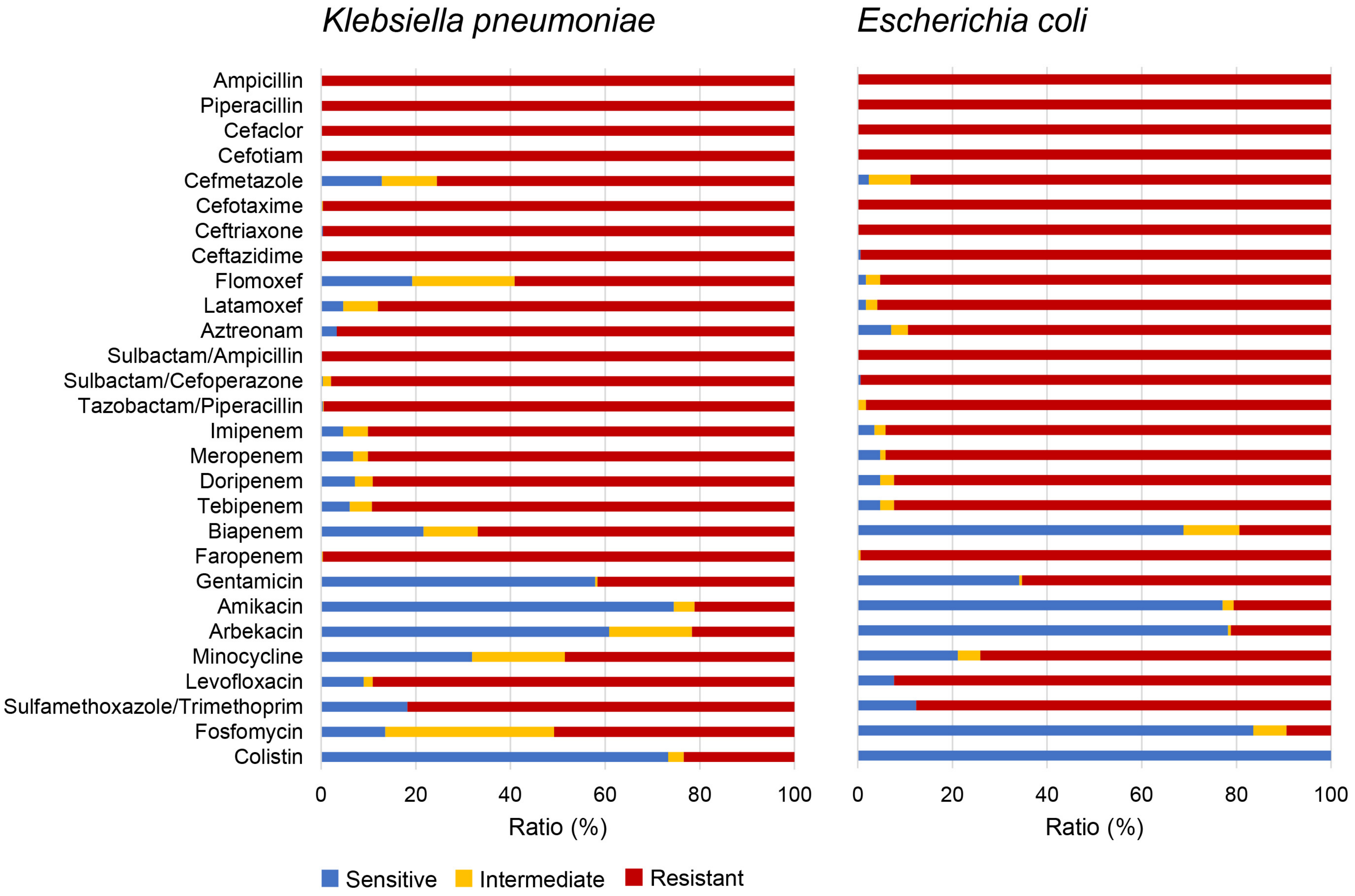

Supplementary Figure 2.

Antibiogram of CRE of each carbapenemase genotype.

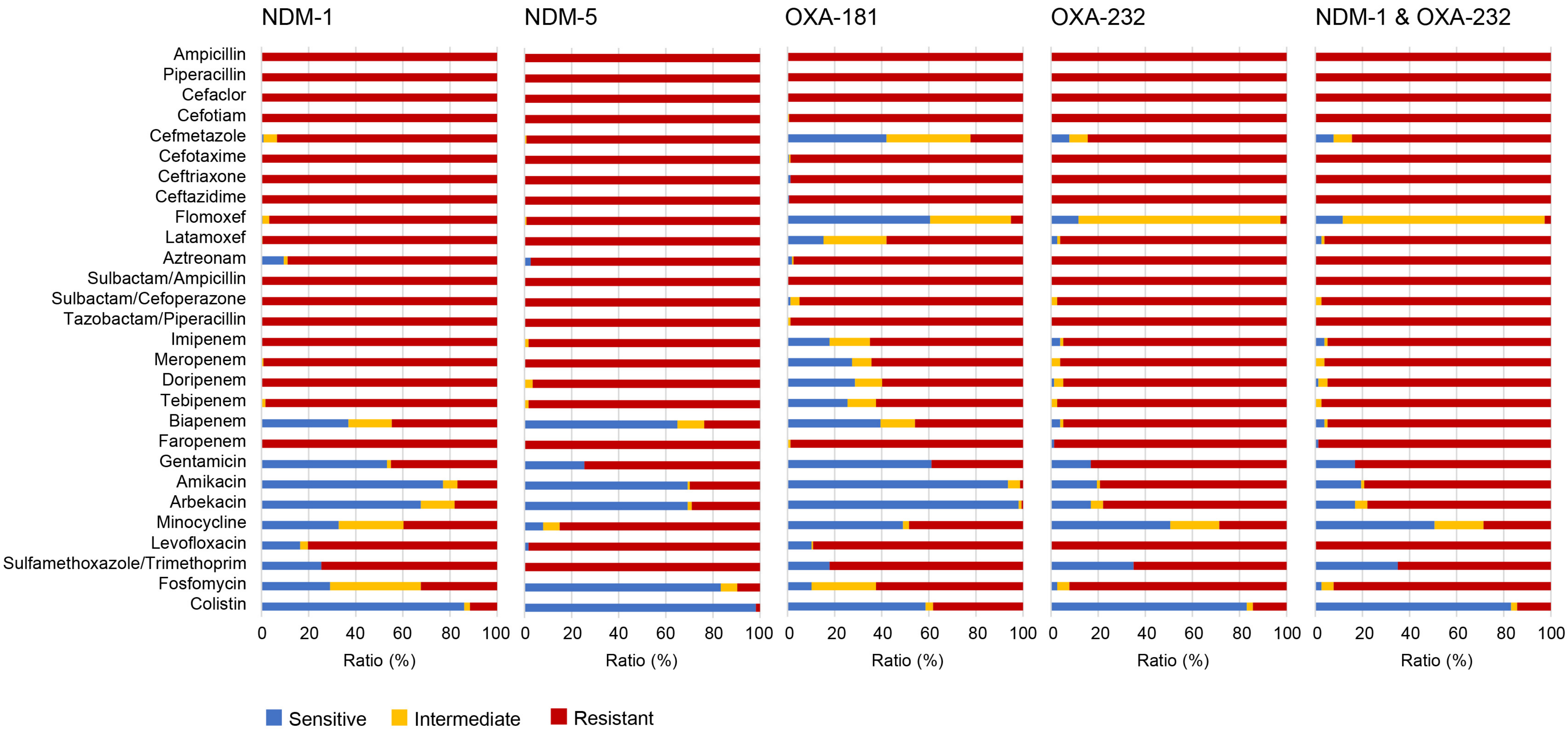

Supplementary Figure 3.

Distribution of carbapenemase genes among all provinces. The number of isolates in each province is shown in parentheses.

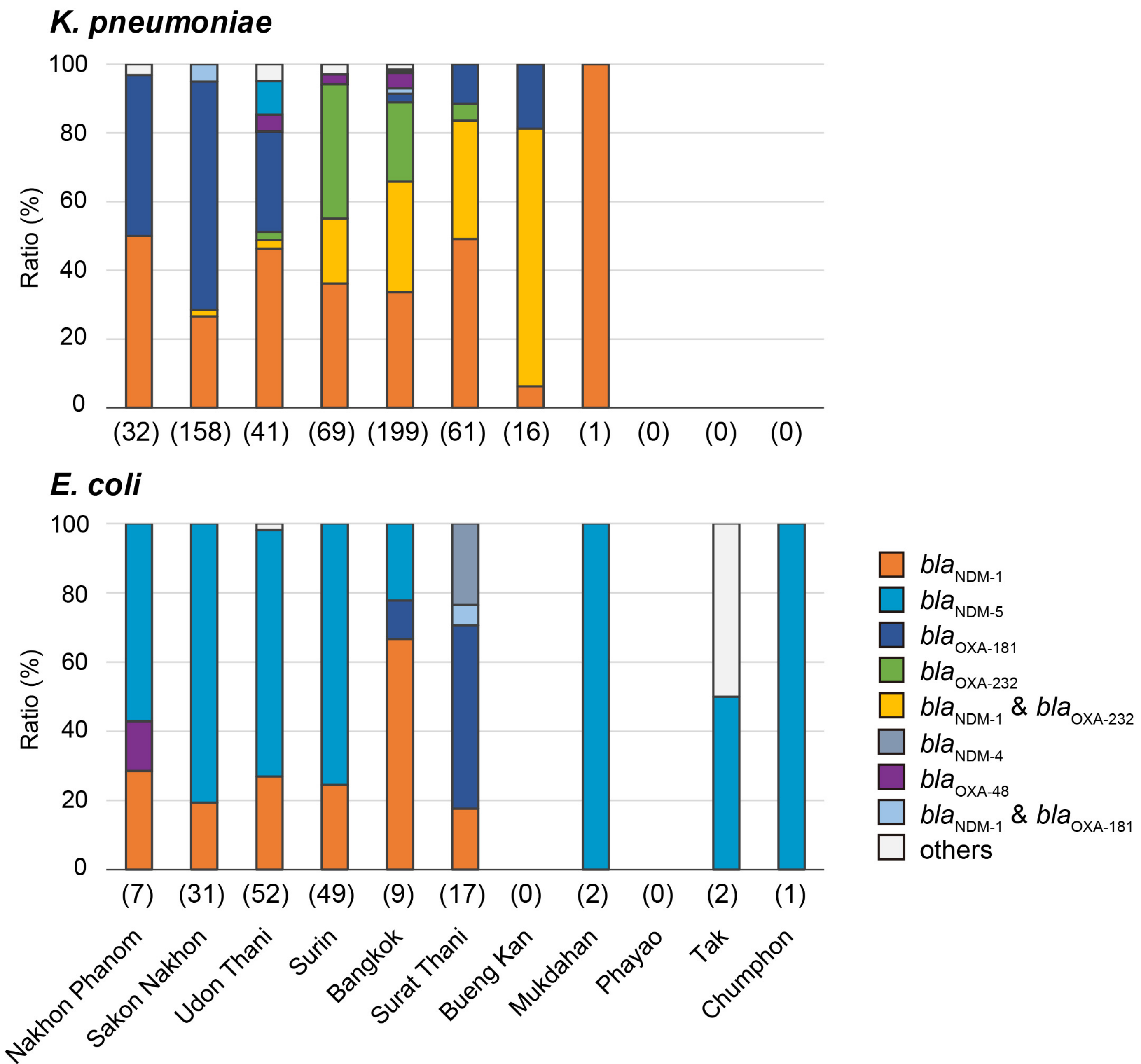

Supplementary Figure 4.

Geographical and sequence type-based distribution of all *bla*$_{NDM-1}$-harbouring plasmid replicon types in isolates with *bla*$_{NDM-1}$. Each coloured cell represents the ratio of the total number of plasmids of the same replicon type. N.D., not determined. BU: Bueng Kan, NP: Nakhon Phanom, SN: Sakon Nakhon, UT: Udon Thani, MU: Mukdahan, SU: Surin, BK: Bangkok, TK: Tak, CP: Chumphon, and ST: Surat Thani.

Ratio (%): 30-100, 20-30, 10-20, 0-10, 0

			Province										*K. pneumoniae*											*E. coli*					
		Total No.	BU	NP	SN	UT	MU	SU	BK	TK	CP	ST	ST11	ST340	ST336	ST231	ST16	ST14	ST147	ST101	ST15	ST48	ST others	ST410	ST361	ST405	ST131	ST167	ST others
**NDM-1**	N2	112	1	9	12	13	1	24	22			30	19	5	6		5		12	12	2	1	20	8		1	4		17
	FIB(pKPHS1)	38		6	26	3			2			1							35		1		2						
	FIB(pQil)_FII(K)	14				2			12							14													
	C	7				7																1	2				1		3
	FIB(pNDM-Mar)_HI1B(pNDM-MAR)	5			1				4									4					1						
	FIB(K)_FII(K)	4		2	2										1							1	2						
	FIB(pQil)_FII(pAMA1167-NDM-5)	4				1			3							4													
	FII(pAMA1167-NDM-5)	3				1		1	1							3													
	FIB(pNDM-Mar)_HI1B(pNDM-MAR)_R	3							3									3											
	C_FII	2				2																							2
	FIB(pQil)_R	2			2														2										
	FIB(pQil)_ColRNAI	2							2										2										
	FIB(K)_N2	2				2									1							1							
	FIB(pQil)	2				1			1										2										
	C_FII(pRSB107)	1			1																				1				
	FII_N2	1										1														1			
	FIB(pQil)_FII(K)_FII(pAMA1167-NDM-5)	1			1											1													
	FIB(K)_FII(K)_N2	1							1						1														
	C_FIA(HI1)	1							1										1										
	FIB(K)	1							1														1						
	FIB(pNDM-Mar)	1							1										1										
	N2_Col440I	1							1					1															
	FIB(pNDM-Mar)_FIB(pQil)_FII(K)_HI1B(pNDM-MAR)	1							1							1													
	C_FII(K)	1							1														1						
	C_FIB(pQil)_FII(K)	1							1							1													
	ND (NDM-1)	33		1	3	1		12	15			1		4		1	7	2	3		5	1	5	2			1		2

Supplementary Figure 5.

Geographical and sequence type-based distribution of all bla_{NDM-5}-harbouring plasmid replicon types in isolates with bla_{NDM-5}. Each coloured cell represents the ratio of the total number of plasmids of the same replicon type. N.D., not determined. BU: Bueng Kan, NP: Nakhon Phanom, SN: Sakon Nakhon, UT: Udon Thani, MU: Mukdahan, SU: Surin, BK: Bangkok, TK: Tak, CP: Chumphon, and ST: Surat Thani.

Ratio (%): 30-100, 20-30, 10-20, 0-10, 0

		Province										*K. pneumoniae*											*E. coli*					
NDM-5	Total No.	BU	NP	SN	UT	MU	SU	BK	TK	CP	ST	ST11	ST340	ST336	ST231	ST16	ST14	ST147	ST101	ST15	ST48	ST others	ST410	ST361	ST405	ST131	ST167	ST others
FII(pAMA1167-NDM-5)_Q1	29			8	4		17																28					1
FIA_FIB(AP001918)_FII(pAMA1167-NDM-5)_Q1	24		1	7	1	1	13			1													24					
FII	12		1	5	6																	1		10				1
Q1	6				3		3																4					2
FIA_FIB(AP001918)_FII(pRSB107)	4			1	3																				3			1
FII_FII(pRSB107)	3			1	2																			3				
FII(pAMA1167-NDM-5)	3		1		1	1																	3					
C	2		1		1																		1	1				
X3	1							1																1				
FII(pRSB107)	1				1																							1
FIA_FIB(AP001918)_FII(pAMA1167-NDM-5)	1				1																		1					
FII_FII(pAMA1167-NDM-5)	1						1																1					
FIA_FIB(AP001918)_FII_Q1	1						1																1					
FIA_FIB(AP001918)_FII(pRSB107)_Col156	1				1																				1			
FIA_FIB(AP001918)_FII_FII(pRSB107)_Col156	1				1																							1
FIB(AP001918)_FII	1				1																					1		
R	1				1																1							
FII(K)	1				1																1							
N.D.	21			3	13		2	2	1			1				1							1	2	10		5	1

Supplementary Figure 6.

Phylogenetic analysis of sequence type (ST)16 of *K. pneumoniae* isolates and distribution of pair-wise SNP distances. Phylogenetic tree was based on 895 chromosomal core genome SNPs. Scale bar indicates the number of nucleotide substitutions per site. Phylogenetic tree was drawn as mid-rooted tree in a circular format, representing the carbapenemase genotype in relation to the province from where the isolates were obtained. Sub-clonal clade is indicated by a black strip in the outermost circle. The bar graph on the right side of the phylogenetic tree shows the distribution of pair-wise SNP distances in clades 6 and 7. Vertical dashed line represents the threshold of clonality for *K. pneumoniae*.

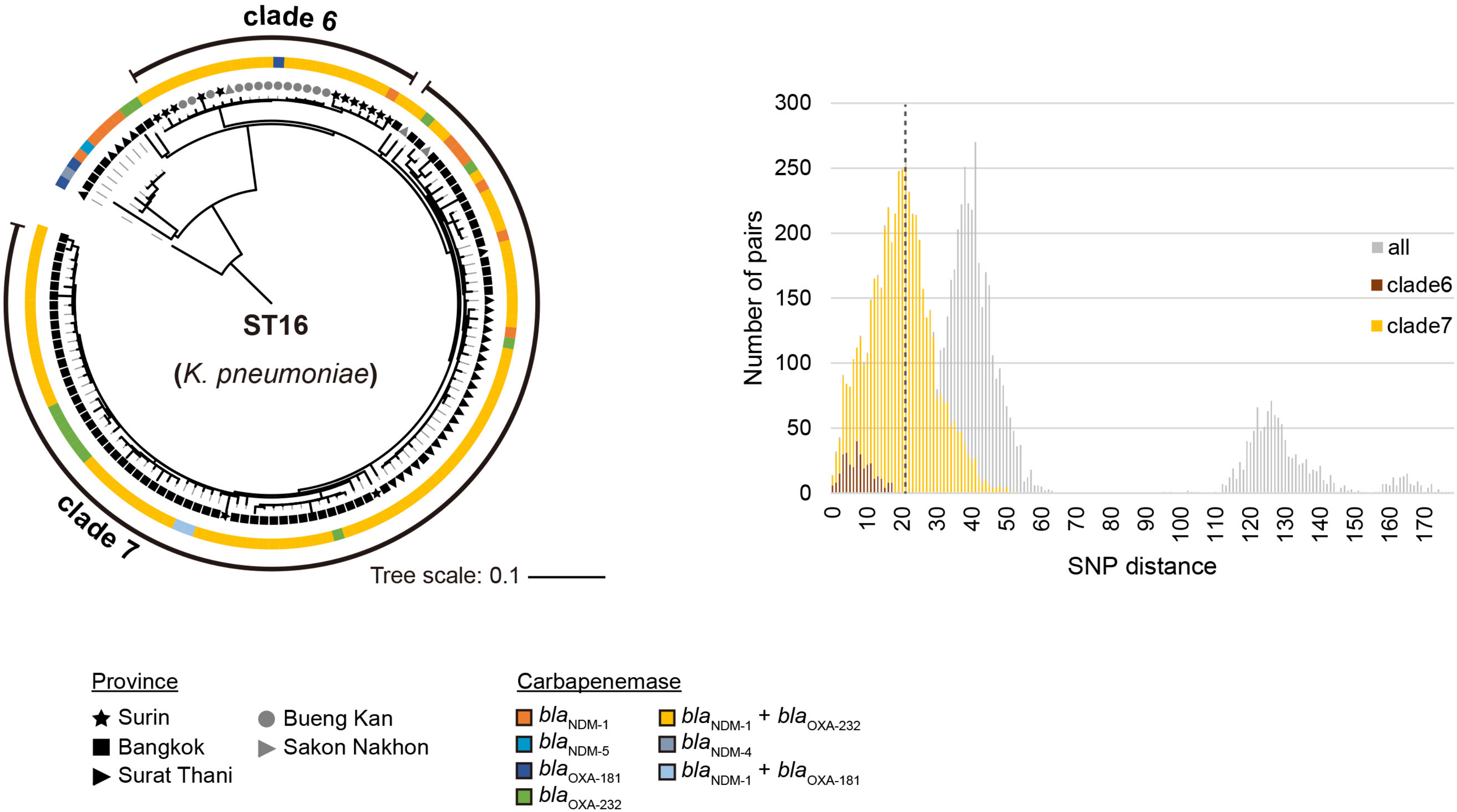

Supplementary Figure 7.

Distribution of two major replicon types of *bla*$_{NDM-5}$-harbouring plasmids in *bla*$_{NDM-5}$-associated sub-clonal clade. Phylogenetic tree is the same as that in Figure 5.

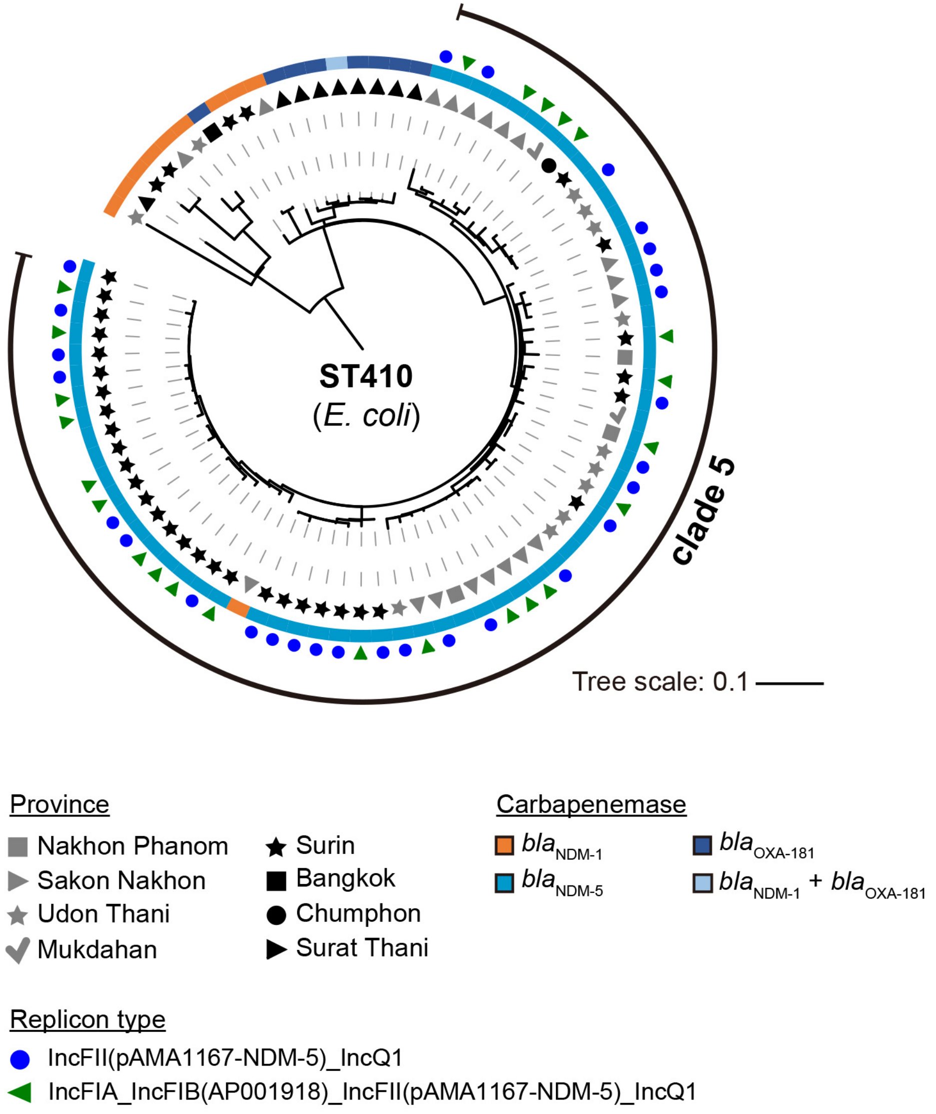

Supplementary Table 1.

Study periods for all provincial hospitals from where samples were collected for this study.

Province	Date_start	Date_end
Sakon Nakhon	Sep-2015	May-2017
Nakhon Phanom	Sep-2015	Jun-2017
Surat Thani	Jan-2015	Aug-2016
Udon Thani	Mar-2015	May-2017
Phayao	Feb-2016	Aug-2017
Tak	Jul-2016	Aug-2016
Chumphon	Jan-2016	Sep-2016
Mukdahan	Nov-2016	Nov-2016
Surin	Apr-2016	Jul-2017
Bueng Khan	Mar-2017	Jun-2017
Bangkok	Mar-2012	Jan-2017

Supplementary Table 2.

Primers used for the detection of carbapenemase genes by PCR.

Gene name	Forward	Reverse
bla$_{NDM}$	5’-GGTTTGGCGATCTGGTTTTC-3’	5’-CGGAATGGCTCATCACGATC-3’
bla$_{OXA}$	5’-GCGTGGTTAAGGATGAACAC-3’	5’-CATCAAGTTCAACCCAACCG-3’
bla$_{KPC}$	5’-CGTCTAGTTCTGCTGTCTTG-3’	5’-CTTGTCATCCTTGTTAGGCG-3’
bla$_{IMP}$	5’-GGAATAGAGTGGCTTAAYTCTC-3’	5’-GGTTTAAYAAAACAACCACC-3’
bla$_{VIM}$	5’-GATGGTGTTTGGTCGCATA-3’	5’-CGAATGCGCAGCACCAG-3’

Supplementary Table 4.

Diversity of carbapenemase genes in terms of geography and STs.

	Geographical diversity		Diversity of STs in KP			Diversity of STs in EC		
**Carba-penemase genotype**	**Number of provinces (% of total)**	**Simpson's diversity (95% CI)**	**Number of STs (% of total)**	**Simpson's diversity (95% CI)**	**STs (Number of isolates)**	**Number of STs (% of total)**	**Simpson's diversity (95% CI)**	**STs (Number of isolates)**
bla$_{NDM-1}$	8 (72.7)	0.81<br>(0.79–0.83)	35 (81.4)	0.88<br>(0.85–0.91)	ST147 (58)<br>ST231 (25)<br>ST11 (19)<br>ST16 (12) *	19 (73.1)	0.92<br>(0.87–0.97)	ST410 (10)<br>ST131 (6)<br>ST354 (4)<br>ST1193 (3) *
bla$_{NDM-5}$	8 (72.7)	0.72<br>(0.68–0.76)	4 (9.3)	0.9<br>(0.58–1)<br>[0.72] ‡<br>(0.4–0.9) ‡	ST48 (2)<br>ST11 (1)<br>ST16 (1)<br>ST1089 (1)	9 (34.6)	0.62<br>(0.53–0.71)	ST410 (64)<br>ST361 (17)<br>ST405 (14)<br>ST167 (5) *
bla$_{OXA-181}$	6 (54.5)	0.53<br>(0.44–0.62)	8 (18.6)	0.65<br>(0.61–0.7)	ST340 (66)<br>ST11 (54)<br>ST336 (16)<br>ST147 (5) *	2 (7.7)	0.36<br>(0.04–0.67)	ST410 (8)<br>ST1340 (2)
bla$_{OXA-232}$	4 (36.4)	0.53<br>(0.45–0.6)	6 (14)	0.39<br>(0.27–0.52)	ST231 (59)<br>ST16 (12)<br>ST11 (2)<br>ST14 (2) *	0 (0)	-	-
bla$_{NDM-1 \&}$<br>*bla*$_{OXA-232}$	6 (54.5)	0.63<br>(0.55–0.71)	2 (4.7)	0.15<br>(0.06–0.23)	ST16 (105)<br>ST14 (9)	0 (0)	-	-

Simpson's diversity indices were calculated with 95% confidence intervals (CI) by approximation method. ‡The bootstrap mean of the index with 95% confidence intervals were additionally calculated because of the small number of isolates. *Results for representative STs. Results for all STs are summarised in Supplementary Table 5.

Supplementary Table 5.

STs of isolates harbouring each carbapenemase genotype.

***Klebsiella pneumoniae*, ST (No. of isolates)**
***bla*$_{\mathbf{NDM-1}}$**
ST147 (58), ST231 (25), ST11 (19), ST101 (12), ST16 (12), ST340 (10), ST336 (9), ST14 (9), ST15 (8), ST48 (5), ST2673 (4), ST334 (2), ST273 (2), ST677 (2), ST477 (2), ST70 (2), ST37 (2), ST120 (1), ST736 (1), ST1859 (1), ST540 (1), ST656 (1), ST39 (1), ST17 (1), ST534 (1), ST1224 (1), ST65 (1), ST307 (1), ST221 (1), ST277 (1), ST323 (1), ST638 (1), ST2133 (1), ST5808 (1), ST2287 (1)
***bla*$_{\mathbf{NDM-5}}$**
ST48 (2), ST1089 (1), ST16 (1), ST11 (1)
***bla*$_{\mathbf{OXA-181}}$**
ST340 (66), ST11 (54), ST336 (16), ST147 (5), ST16 (3), ST17 (1), ST101 (1), ST120 (1)
***bla*$_{\mathbf{OXA-232}}$**
ST231 (59), ST16 (12), ST14 (2), ST11 (2), ST5819 (1), ST147 (1)
***bla*$_{\mathbf{NDM-1}}$ & *bla*$_{\mathbf{OXA-232}}$**
ST16 (105), ST14 (9)
***Escherichia coli*, ST (No. of isolates)**
***bla*$_{\mathbf{NDM-1}}$**
ST410 (10), ST131 (6), ST354 (4), ST1193 (3), ST10 (2), ST88 (2), ST405 (2), ST2011 (2), ST38 (2), ST8891 (1), ST2144 (1), ST1236 (1), ST48 (1), ST4219 (1), ST3910 (1), ST744 (1), ST1722 (1), ST361 (1), ST457 (1)
***bla*$_{\mathbf{NDM-5}}$**
ST410 (64), ST361 (17), ST405 (14), ST167 (5), ST46 (3), ST10210 (3), ST2659 (1), ST34 (1), ST131 (1)
***bla*$_{\mathbf{OXA-181}}$**
ST410 (8), ST1340 (2)

Supplementary Table 6.

Representative plasmids reconstructed from long-read sequencers.

**Plasmid name**	**Sequencer**	**Assembler**	**Replicon type**	**Carbapenemase genotype**	**Plasmid size (bp)**	**DDBJ accession number**	**Similar plasmid (GenBank accession no.)**
pC057_NDM1	PacBio	HGAP	IncN2	*bla*$_{NDM-1}$	41181	LC521837	KJ413946.1
pC435_NDM1	GridION	Unicycler	IncFIB(pQil)	*bla*$_{NDM-1}$	54064	LC521845	AP018834.1
pC048_NDM1	GridION	Unicycler	IncFIB(pQil)_IncFII(K)	*bla*$_{NDM-1}$	152756	LC521835	KF295829.1
pKP164_NDM1	PacBio	HGAP	IncFIA_IncFIB(AP001918)_IncFII_IncFII	*bla*$_{NDM-1}$	116295	LC521851	CP024039.1
pKP100_NDM1	PacBio	HGAP	IncFIB(pNDM-Mar)_IncHI1B(pNDM-MAR)	*bla*$_{NDM-1}$	296678	LC521849	AP018748.1
pC070_NDM1	PacBio	HGAP	IncFIB(pQil)_IncR	*bla*$_{NDM-1}$	92204	LC521839	none
pC405_NDM5	GridION	Unicycler	IncFIA_IncFIB(AP001918)_IncFII_IncQ1	*bla*$_{NDM-5}$	108173	LC521844	CP026474.1
pC063_NDM5	PacBio	HGAP	IncFII	*bla*$_{NDM-5}$	102015	LC521838	AP018147.1
pC281_NDM5	GridION	Unicycler	IncFIA_IncFIB(AP001918)_IncFII(pRSB107)	*bla*$_{NDM-5}$	156037	LC521842	MN218686.1
pEC08_NDM5	GridION	Unicycler	IncX3	*bla*$_{NDM-5}$	61114	LC521848	KY041843.1
pC278_NDM5	GridION	Unicycler	IncFIB(AP001918)_IncFII	*bla*$_{NDM-5}$	152332	LC521841	CP027703.1
pKP161_OXA232	GridION	Unicycler	ColKP3	*bla*$_{OXA-232}$	6141	LC521850	CP050165.1
pC005_OXA181	PacBio	HGAP	IncX3_ColKP3	*bla*$_{OXA-181}$	51463	LC521833	AP018831.1
